# 3D multi-phase balanced non-steady-state free precession acquisition for multi-parameter mapping

**DOI:** 10.1007/s10334-025-01262-2

**Published:** 2025-05-26

**Authors:** Riwaj Byanju, Gyula Kotek, Mika W. Vogel, Stefan Klein, Juan A. Hernandez-Tamames, Dirk H. J. Poot

**Affiliations:** 1 Department of Radiology and Nuclear Medicine, Erasmus MC, Rotterdam, The Netherlands; 2 GE Healthcare, Hoevelaken, The Netherlands

**Keywords:** Transient imaging, SSFP, qMRI, Subspace-constrained, Extended readout

## Abstract

**Objective:**

This study presents the 3D MP-b-nSSFP sequence for multi-parametric mapping.

**Methods:**

We evaluate several aspects of the 3D implementation, like the type of RF pulse (selective/non-selective), the readout duration, the undersampling pattern, and the acceleration factor. We use undersampled scans with subspace-constrained reconstruction and extended spiral readouts to achieve clinically acceptable scan times. The repeatability and accuracy of the $T_1$ and $T_2$ maps are compared with a reference technique in phantom and three volunteer scans.

**Results:**

Compared with selective refocusing pulses, we observe lower bias with non-selective pulses, despite modeling the spatially varying effect of the pulses in the fitting process. $T_1$ and $T_2$ maps obtained from phantom scans were comparable to the nominal values and those from reference scans. $T_1$ values in vivo were underestimated compared to the reference scan. The maps with an acquisition matrix of $256 \times 256 \times 44$ and resolution $1 \times 1 \times 3$ $\text {mm}^3$ were acquired in 11 min.

**Conclusion:**

We show that 3D MP-b-nSSFP can be used for multi-parameter mapping within clinically acceptable scan time. Phantom scans show results in good agreement with reference scan results. However, the in vivo scan underestimated $T_1$.

## Introduction

Quantitative MRI (qMRI) has the potential to increase the diagnostic information obtained from MR acquisitions by achieving comparable and repeatable scans across scanners and sequences by measuring tissue parameters such as $T_1$ and $T_2$ [1]. Traditionally, qMRI parameters are estimated by fitting the signal evolution across each voxel to a parametric model. The MR signal is influenced by imperfections of the MR scanner that are scan-specific such as magnetic field inhomogeneities ($B_0$) and radio-frequency imperfections ($B_1$) [2]. Conventional qMRI sequences are based on the steady-state response of the MR, where the effect of such scan-specific components can be minimized. However, these take impractically long scan times and have found limited clinical applications [3].

Recently, quantitative MRI techniques based on the transient response of magnetization have been developed [4–23]. These approaches use various specific sequences of RF pulses and gradients to drive the magnetization through a sequence of states that are imaged and together allow the simultaneous mapping of multiple tissue parameters and often scanner-specific parameters such as $B_0$ and $B_1$ as well. Approaches such as Ref. [24] use a hybrid state which combines a quasi steady-state transversal magnetization with a non-steady-state longitudinal magnetization. Many of these approaches, including MR fingerprinting, use pattern-matching algorithms to identify tissue parameters by comparing measured signals with simulated signal dictionaries, while other methods effectively used non-linear inversion [24–26].

In addition, steady-state methods have advanced and can achieve efficient multi-parametric quantification [27–29]. Various sequences have further expanded the capability to characterize additional tissue properties such as chemical exchange saturation transfer (CEST) [30–32], fat–water separation [33–35], and arterial spin labeling [36, 37]. Numerous acceleration strategies have also been investigated to reduce acquisition times [12, 23, 38–40]. For a broader overview of recent developments in quantitative MRI methods, the reader is referred to comprehensive review articles [41, 42].

Dictionary matching allows the approximate solving of highly non-convex optimization problems that often arise in such sequences. However, due to the lack of analytical expression describing the signal, finding optimal acquisition settings suh as repetition time (TR) and flip angles (FA) for such an approach has been an active field of research [41]. For instance, Zhao et al. [43] and Asslander et al. [24] use the Cramér Rao lower bound (CRLB) for finding optimal acquisition settings for a transient sequence; however, the optimization problem is non-convex and dependent on initialization. Fuderer et al. [44] present another way to find optimal settings. Their approach is based on block analysis of a K-space-domain Jacobian which works with a predefined phase-encoding pattern. Kotek et al. [3] proposed a novel approach called multi-phase balanced, non-steady-state free precession (MP-b-nSSFP) where the transient magnetization response of repetitive blocks consisting of RF and gradient pulses can be expressed as an algebraic description that allows an analytical solution. The analytical solution simplifies in-silico optimization of acquisition settings. Using the CRLB, an optimized sequence setting was designed for producing $T_1$ and $T_2$ along with $B_0$ and $B_1$ maps.

This work aims to extend that novel MP-b-nSSFP method to 3D while maintaining a clinically acceptable scan time. A 3D acquisition requires additional slice encoding gradients, which involves various choices and demands adjustments to the reconstruction pipeline. In addition, with a naive, complete k-space sampling, the acquisition time for a 3D scan would be prohibitively long. Hence, we investigate the possibility of an undersampled scan to reduce acquisition time by exploiting redundancies across the contrasts. We use subspace-constrained reconstruction (SCR) to leverage the redundancy across signal evolution. Moreover, we use an extended readout to cover more k-space positions in a shorter time and use a $B_0$ compensation step to remove the effect of $B_0$ inhomogeneities due to the extended readout.

## Theory

In this section, we start by describing the previously proposed 2D MP-b-nSSFP [3], followed by steps to extend the method to scan in 3D.

### 2D MP-b-nSSFP

**Fig. 1:**
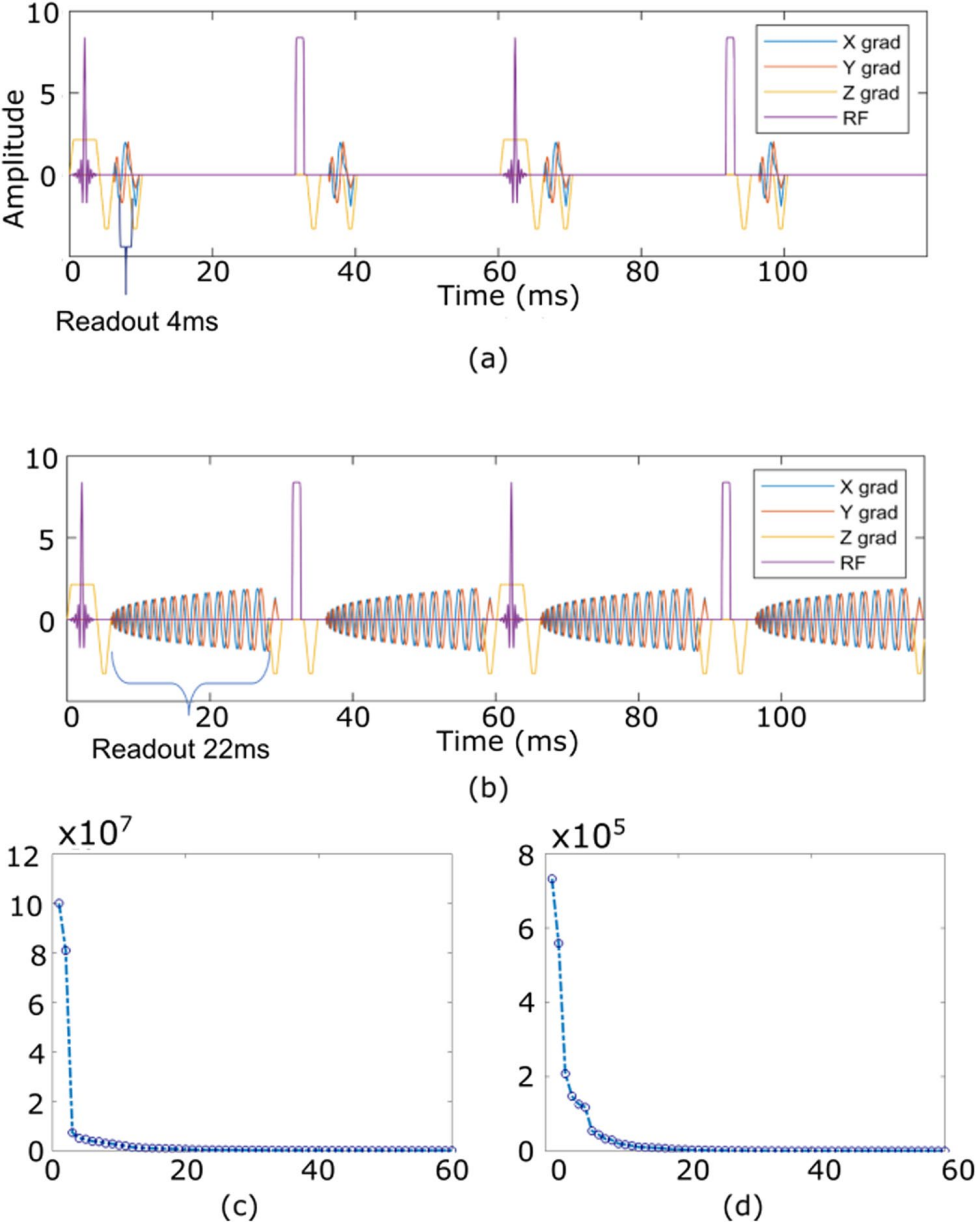
An optimized block of four RF pulses $30^{\circ }_{x}, 175^{\circ }_{y},30^{\circ }_{y}, 175^{\circ }_{x}$. **a**, **b** The block is repeated 12 times in a pulse train (48 spiral readouts) and a delay of 3 s was applied after every pulse train with readout length **a** 4 ms and **b** 22 ms. **c**, **d** Energy distribution across number of subspace components computed from **c** Si and **d** Mu dictionary for slice 22

The previously proposed 2D MP-b-nSSFP [3] used a train of pulses consisting of 25 repetitions of an optimized block of 4 RF pulses $30^{\circ }_{x}, 175^{\circ }_{y},30^{\circ }_{y}, 175^{\circ }_{x}$ with a readout after every RF pulse as shown in Fig. 1a. The time between RF pulses ($\text {TRp}$) was 30 ms; hence, a single block took 120 ms, the total train was 3000 ms and there was a delay of 3000 ms between pulse trains. The $30^{\circ }$ RF pulses were high-fidelity Shinnar–Le Roux (SLR) algorithm-based slice selective pulses with a time-bandwidth product of 16 to avoid within-slice spin dispersion [45]. Data were acquired with spiral readouts of 4 ms without acceleration. The readouts measured after each RF pulse in a train are considered different contrasts. Thus, the sequence had 100 different contrasts. Six qMRI parameters: the real and imaginary part of apparent proton density $\Re [I_{0}]$, $\Im [I_{0}]$, $T_1$, $T_2$, $B_{0}$, and $B_{1}$, represented by $\varvec{\theta } \in \mathbb {R}^{6 \times n^v}$ were computed for $n^v$ voxels. SENSE reconstruction [46] was used to produce contrast images. An analytical description of the signal evolution was demonstrated which was used to extract qMRI parameters from the contrast images [3]. Alternatively, a dictionary matching approach [14], where simulations of the Bloch equation were used for dictionary generation, was also used for parameter mapping from the reconstructed contrast images [3]. Note that the Bloch simulation used for dictionary generation used a single spin for a voxel. In the sections below, we refer to this dictionary as ‘single spin dictionary’.

### 3D MP-b-nSSFP

#### Selection of RF pulses

To extend the method to 3D, the slice thickness of the SLR pulses needs to be increased to the full slab width. Having non-selective inversion pulses may cause out-of-slab excitation, creating imaging artifacts [47]. However, slab-selective inversion pulses of sufficiently short duration have varying inversion efficiency for different positions in the slab direction, and hence for the different slices in the slab. Due to this varying inversion efficiency, the signal evolution over the pulse train differs for different slices, even when voxels in those slices have identical tissue properties. This, in turn, may cause a slice-dependent bias in the parameter maps, unless the varying inversion efficiency is taken into account during parameter mapping. To identify the relative importance of these aspects, we evaluate both options. Note that in this work, we used 12 repetitions of RF pulses instead of 25 as in the case of 2D MP-b-nSSFP. We consider this sufficient as the decay of the signal is faster than $T_1$ decay, and hence, for tissues, it is basically fully decayed after 12 repetitions, which take 1440 ms (12  $\times$  4  $\times$   30 ms).

#### Trajectory selection

3D scans can be performed with trajectories such as cartesian, spiral or radial spokes; we focus on spiral readouts because they efficiently encode k-space [48]. It is possible to extend 2D spiral readout to 3D with two main strategies [48]. The first approach is known as a rotating spiral [49], where the plane of the spiral is rotated along the slice encoding dimension. The rotating spiral approach gives naturally isotropic sampling across all dimensions without the possibility to have a different resolution or FOV in the slice direction. However, in practice, often a different resolution and FOV is chosen in the slice direction, allowing scan time to be saved. The ‘stack-of-spirals’ approach allows such adaptation by shifting the spiral k-space trajectories in the slice encoding direction [50]. The stack-of-spiral approach has an advantage in the reconstruction step, particularly when the slice encoding direction is fully sampled. A single inverse Fourier transform is sufficient to separate the signals along the slice encoding dimensions. Then each slice in the stack can be reconstructed using a 2D reconstruction algorithm. In this work, we used stack-of-spiral trajectory with full sampling of the slice direction and reconstructed slices separately.

To perform undersampled scans, we skip different spirals along the contrast dimension. We select the spiral sampled in each contrast by rotating the spiral using the angle given by $\lfloor n^{\text {s}} \times (2\times \phi )\rceil$ where $n^{\text {s}}$ is number of spiral arms and $\phi$ represents golden ratio [48].

#### Subspace-constrained reconstruction for acquisitions with extended readout

Previously, it was demonstrated that spiral readouts in the MP-b-nSSFP sequence could be extended to cover more k-space positions [14]. The optimized block with longer readouts is shown in Fig. 1b. Increasing readouts to approximately 22 ms from 4 ms allows covering the same k-space extend with fewer readouts. However, these extended readouts contain larger effects of $B_0$ inhomogeneities, often leading to blurring artifacts in the reconstructed images [51]. Valenberg et al. [14] indicated that these artifacts could be eliminated during reconstruction using a separate off-resonance map $\varvec{\Delta } \in \mathbb {R}^{n^v}$ map.

Let the tensor $\varvec{M}^{'}$ represent magnetization and subscripts $q = [1, n^{q}]$, $v = [1, n^{v}]$, and $p = [1, n^{p}]$ represent contrast, voxels, and readout, respectively. In the presence of ${B_0}$ inhomogeneity, $\varvec{M}^{'}$ be approximated by:

1 $$\begin{aligned} {M}^{'}_{q,v,p} = {M}_{q,v} e^{-i{\Delta }_{v}\tau _{p}} \end{aligned}$$

where $\tau _{p}$ is the time offset with respect to the magnetization state ${M}_{q,v}$. Note that $e^{-i{\Delta }_{v}\tau _{p}}$ only models the effect of $B_0$ inhomogeneties during readout. A dictionary of echo signals can be created by computing $\varvec{M}$ for a set of tissue property vectors $\varvec{\theta }$. Following the principle of SCR [52], Eq. 1 can be approximated by two singular value decompositions (SVD). The first describes the contrast variations among the echoes with a SVD truncated to $d_1$ components:

2 $$\begin{aligned} \varvec{M} \approx \varvec{U} \varvec{S} \varvec{V^{H}} = \left[ \sum _{m} {{U}_{q, m}} {{S}_{m,m}} {{V^{H}_{v,m}}} \right] _{q, v} \end{aligned}$$

where $\varvec{V^{H}}$ represents transpose of the matrix $\varvec{V}$, $\varvec{U} \in \mathbb {C}^{n^q \times d_1}$, $\varvec{S} \in \mathbb {R}^{d_1 \times d_1}$, $m = [1, d_1]$ and $\varvec{V} \in \mathbb {C}^{n^v \times d_1}$. Note that $d_1$ is chosen empirically. Using conventional SCR, the component images, given by $\varvec{\delta } = \varvec{S}\varvec{V^{H}}$, can be computed using the following optimization problem:

3 $$\begin{aligned} \hat{\varvec{\delta }} = \arg \min _{\varvec{\delta }} \sum _{q,c,p} \Vert {{Y}_{q,c,p} - \sum _{m,v} \mathcal {F}_{q,p,v} {C_{c,v}} {U_{q,m}} {\delta }_{m, v}} \Vert ^2 \quad \end{aligned}$$

where $\varvec{Y} \in \mathbb {C}^{n^q \times n^c \times n^p}$ is the acquired data for each contrast, channel, and sampled k-space location, $\varvec{C} \in \mathbb {C}^{n^c \times n^v}$ represents coil sensitivity maps with $n^c$ is number of coils, $\mathcal {F}$ represents a operator that applies the non-uniform Fourier transform and selects the sampled *k*-space trajectories in an undersampled scan. Note that $\mathcal {F}$ has index *q* as the sampled k-space positions can vary across contrasts. The second SVD describes the phase evolution in presence of $B_0$ inhomogeneities. Considering the first $d_2$ singular values where $d_2$ is chosen empirically, it is given by:

4 $$\begin{aligned} \left[ e^{-i\varvec{\Delta }_{v}\tau _{p}} \right] _{p, v} \approx \varvec{\tilde{U}} \varvec{\tilde{S}} \varvec{\tilde{V}^{H}} = \left[ \sum _{w} {\tilde{U}_{p, w}} {\tilde{S}_{w,w}} \tilde{V}^{H}_{v,w} \right] _{p, v} \end{aligned}$$

where $\varvec{\tilde{U}} \in \mathbb {C}^{n^p \times d_2}$, $\varvec{\tilde{S}} \in \mathbb {R}^{d_2 \times d_2}$, $\varvec{\tilde{V}} \in \mathbb {C}^{n^v \times d_2}$ and $w \in [1, n^{d_2}]$. The component images, $\varvec{\sigma } \in \mathbb {C}^{d_1 \times n_v}$ with $B_0$ inhomogeneity correction can be estimated by solving the modified SCR problem (see Appendix 5 for the derivation):

5 $$\begin{aligned} \hat{\varvec{\sigma }} = \arg \min _{\varvec{\sigma }} \sum _{ q, c, p} \Vert {Y}_{ q, c, p} - \sum _{ m, w, v} {\tilde{U}}_{ p, w} {\tilde{S}}_{ w, w} U_{ q, m} \mathcal {F}_{q, p,v} \varvec{C}_{c, v} \tilde{V}^{H}_{ v, w} {\sigma }_{ m, v} \Vert ^2 \quad . \end{aligned}$$

Equation 5 is solved with conjugate gradient algorithm [14]. Finally, the contrast images can be computed as $\varvec{\hat{I}} = \left[ \sum _{m}{U}_{q, m}{\hat{\sigma }_{m, v}}\right] _{q,v}$.

Similarly, $B_0$ compensation can also be incorporated into *SENSE* reconstruction [46] by modifying the SENSE optimization problem:

6 $$\begin{aligned} { \hat{\varvec{I}} = \arg \min _{\varvec{I}} \sum _{q,c,p} \Vert {{Y}_{q,c,p} - \sum _{w,v}{\tilde{U}}_{p,w}{\tilde{S}}_{w, w} \mathcal {F}_{q,p,v} \varvec{C}_{c,v} {{\tilde{V}}^{H}_{v, w}} {I}_{q,v}} \Vert ^2 \quad }. \end{aligned}$$

Note that different from conventional SENSE optimisation problem, SVD components from the $B_0$ compensation term in Eq. 1 are present in Eq. 6, and also note that Eq. 6 can be optimized for each *q* independently.

#### QMRI parameter mapping

To fully simulate the effect of the pulse sequence on the spins, the dictionary for qMRI mapping from reconstructed images was created using the actual gradient and RF waveforms in the Bloch simulation. Unlike the previous work on 2D MP-b-nSSFP, 5000 sub-spins distributed along slice encoding direction over 1.8 times the slab width were used. Note that the slab profile is automatically encoded by the simulating the RF and gradient pulses for all sub-spins. The (balanced) z-encoding gradients were treated separately so that one run of the Bloch simulation creates the signal for all z-encodes so that with subsequent inverse Fourier transform, the signal for each slice position is obtained. Though accurate, this simulation is computationally too expensive to be used directly for fitting. Hence, for each slice position *z*, we constructed a dictionary [4] of signal evolutions, computed for a grid of $\varvec{\theta }$. In the next sections, we refer to this dictionary as ’multiple spin dictionary’.

#### Abbreviations

As there are multiple options in the method, we create three term names to differentiate the choices. Specifically, the first term specifies RF pulse type: ‘*sRF*’ for selective and ‘*nsRF*’ for non-selective. The second term specifies the reconstruction technique: ‘*SENSE*’ for individual echoes reconstructed using *SENSE* (Eq. 6), ‘*SCR*’ for joint reconstruction using SCR (Eq. 3). The third term specifies the dictionary used for mapping: ‘*Si*’ for single-spin assumption and *Mu* for multiple-spins dictionary. For example, the notation “*nsRF-SENSE-Mu*” represents non-selective inversion pulse-based acquisition reconstructed with *SENSE* using the assumption of multiple spins per voxel in the generation of the dictionary. Note that while the choice between the RF pulses and the dictionaries have to be investigated, there are clear reasons for choosing between the reconstruction techniques. The *SCR* reconstruction technique is introduced to allow reconstruction of higher acceleration factors than those achievable by *SENSE*. *SENSE* will be used only for acceleration factor 2 reconstructions so that the reconstruction step is independent of the choice of the dictionary and will be treated as reference when used.

## Experiments

We performed three phantom experiments and performed scans on three healthy volunteers to investigate various aspects of the 3D-MP-b-nSSFP scans. We first describe common experimental details and define some abbreviations followed by the experiments.

$$B_0$$ ***compensation***

For $B_0$ compensation, a $\varvec{\Delta }$ map is needed. In previous work by Van Valenburg et al. [14], a separate scan was used to record the $\varvec{\Delta }$ map. In this work, we use a two-step approach; we first perform SCR without compensation, given in Eq. 3, followed by the parameter mapping step to get the initial $\varvec{\Delta }$ map. This $\varvec{\Delta }$ map wraps around if $|\varvec{\Delta }\text {TRp}| >\pi$. To unwrap, the algorithm in Appendix 1 is used. After unwrapping, the map $\varvec{\Delta }$ is used for SCR by Eq. 11 to get $B_0$ compensated images.

***Dictionary generation for SCR and qMRI parameter mapping***

Two dictionaries were created for a grid of $\varvec{\theta }$ values by varying $T_1$ and $T_2$ logarithmically between 100–2500 ms and 20–300 ms, respectively. Similarly, $\varvec{\Delta }$ and $B_1$ linearly within the ranges of $-$ 200 to 200 Hz and 0.8–1.2, respectively. The real and imaginary part of apparent proton density are set as linear parameters and are scaled during the fitting. For *SCR* reconstructions, the number of subspace components ($d_1$) was 12, which as shown in Fig. 1b, c accounts for about $99\%$ of the signal evolution for Mu dictionary.

***Dictionary matching technique for qMRI parameter mapping***

The computationally efficient dictionary matching algorithm proposed by van Valenberg et al. [14] was used. According to this algorithm, the final parameters are obtained with dictionary interpolation-based fitting after initially matching the observed signal evolutions to the dictionary. For each dictionary individually, the number of steps in each parameter was automatically selected to reach an acceptable predicted interpolation accuracy [14].

***Coil sensitivity computation***

Coil sensitivity maps were computed from data of a separate Multiple Gradient Echo scan using NUFFT-based ESPIRiT [53].

### Phantom scan experiments

The following three experiments aim to choose between the best acquisition settings and parameter mapping techniques. Then the repeatability of the chosen combination is tested and compared with another reference technique in terms of accuracy.

We performed three experiments with the Eurospin [54] phantom on a GE 1.5 T Artist scanner using a 20-channel head and neck coil and acquisition settings listed in Table 1. Note that due to (possibly too conservatively) predicted gradient heating, the maximum slew rate had to be reduced to 30 $\text {T/m/s}$, thereby reducing bandwidth as well as k-space coverage by the extended spiral readout.

#### Phantom experiment I

The first phantom experiment compares selective and non-selective inversion pulses as well as the choice between *Si* and *Mu*. Hence, two 3D MP-b-nSSFP acquisitions with selective and non-selective inversion pulses are acquired. The acceleration factor was limited to 2, and *SENSE* reconstruction was used for both. Therefore, the comparison was between *sRF-SENSE-Si*, *sRF-SENSE-Mu*, *nsRF-SENSE-Si*, and *nsRF-SENSE-Mu*. Accuracy of the four sets of 3D parameter maps was assessed by comparing to the nominal values provided in the data sheet of the phantom. Specifically, a 3D ROI is drawn for each tube in which, for each slice separately, the mean and standard deviation of $T_1$ and $T_2$ are computed.

**Table 1:** Common acquisition setting for phantom and volunteer scans

Scan settings	Acquisition matrix	Resolution (mm$^3$)	Acceleration factor	Scan time (rounded off to minutes)	No. of contrasts
3D MP-b-nSSFP	$256 \times 256 \times 44$	$1 \times 1 \times 3$	2/4/5.33 / 8	30/15/11/8	48
Multiple-gradient-echo	$256 \times 256 \times 44$	$1 \times 1 \times 3$	2	1	16
MAGIC	$256 \times 256 \times 44$	$1 \times 1 \times 3$	3	7	NA

#### Phantom experiment II

To assess acceleration performance, we obtain 3D MP-b-nSSFP with acceleration factors of 2, 4, 5.33, and 8. Based on the phantom experiment I results, we select the best-performing RF pulse. We reconstruct the data using *SENSE* for the factor 2 acceleration, which serves as a reference for comparison with higher acceleration factors. For the other acceleration factors, we use *SCR* and the subspace generated from the best-performing dictionary in phantom experiment I for reconstruction. Parameter mapping uses the same dictionary.

To evaluate the accuracy of $T_1$ and $T_2$ values, we compare the mean and standard deviation of $T_1$ and $T_2$ within the ROIs to the nominal values of the phantom [54].

#### Phantom experiment III

Phantom experiment III was performed with the following aims:

- To examine the repeatability of the 3D MP-b-nSSFP.
- To compare the accuracy of $T_1$ and $T_2$ maps produced by 3D MP-b-nSSFP to a reference sequence.

We perform a test-retest scan on the phantom using the best-performing RF pulse, reconstruction, and parameter mapping technique in phantom experiment I and the highest acceleration factor that produces artifact-free maps and has an acceptable loss in accuracy in phantom experiment II.

We obtained a reference map using the Magnetic Resonance Image Compilation (MAGIC) product sequence. This sequence involves a multi-echo acquisition of a saturation-recovery combined with a Fast Spin-Echo sequence called QRAPMASTER [55], which allows us to obtain information on $T_1$, $T_2$, and proton density. The MAGIC scan was conducted with an acceleration factor of 3, and the resolution and field of view matched the 3D-b-nSSFP scan as shown in Table 1 . To evaluate the accuracy of $T_1$ and $T_2$ values, we compared the mean and standard deviation of $T_1$ and $T_2$ within the ROIs to the nominal values of the phantom [54] for both reference and 3D MP-b-nSSFP.

### Volunteer scans

We performed two experiments to compare 3D MP-b-nSSFP with a reference technique on volunteers. The volunteer scans were performed in a study approved by our institutional review board (*Medische Ethische Toetsings Commissie Erasmus MC*, protocol number 2014-096). Before participating, all subjects provided informed consent.

#### Volunteer scan experiment I

Three healthy volunteers were scanned using the same scanner and coils as in the phantom experiments. We performed the 3D MP-nSSFP with acceleration factor 5.33 and MAGIC scan with acceleration factor 3 using the same scan settings as in phantom experiment III.

To observe the effect of $B_0$ compensation, a special reconstruction of *nsRF-SCR-Mu* is performed without $B_0$ compensation and this is compared to the otherwise used reconstructions with $B_0$ compensation. The parameter maps were obtained from 3D MP-nSFFP data using *nsRF-SCR-Mu*. Parameter maps from the MAGIC scan were obtained using online reconstruction in the scanner. The synthetic $T_1$ weighted images from MAGIC were used in Freesurfer to perform subregion analysis [56]. Out of the 39 segments, the 9 segments with the largest volume were used as ROIs: cerebral white matter, cerebral cortex, cerebrospinal fluid (CSF), cerebellum white matter, cerebellum cortex, thalamus, caudate, putamen, and pallidum. CSF is known to have $T_1$ and $T_2$ values outside the accurate range for most qMRI sequences, so it is omitted in the analysis.

To compensate subject displacements between the , a synthetic $T_1$ weighted image was created from the 3D MP-b-nSSFP maps and registered to the synthetic $T_1$ weighted image of MAGIC using Linear Image Registration Tool from FMRIB [57]. Subsequently, the found transformation was applied to the 3D MP-b-nSSFP maps, using linear interpolation. To compare the results between 3D MP-b-nSSFP and MAGIC, the distribution of parameter values in each ROI of the maps is shown as box plot.

#### Volunteer scan experiment II

To investigate the repeatability of 3D MP-b-nSSFP on in vivo scans and compare the repeatability in comparison with MAGIC, we performed test–retest scans on a additional volunteer in two different scan sessions performed 10 days apart. The scanner, coils, and scan settings were same as volunteer scan experiment I. The parameter mapping as well as the subsequent segmentation and registration were performed similarly to volunteer scan experiment I. Note that, the registration was performed between 3D MP-b-nSSFP and MAGIC results from the same session, and test–retest data was not registered.

**Fig. 2:**
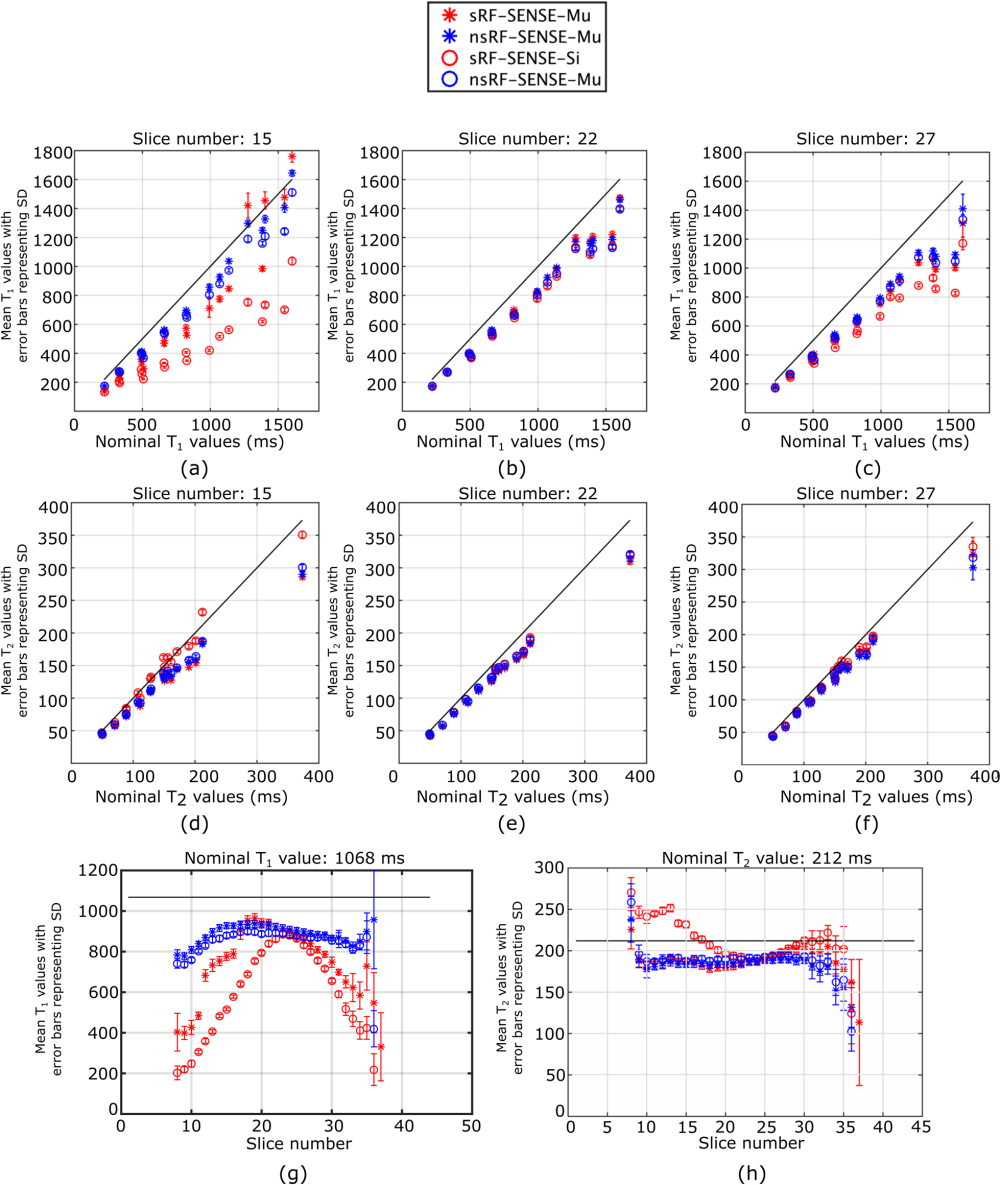
Results of phantom experiment I. The results from *sRF-SENSE* are shown in red color and *nsRF-SENSE* are shown by blue markers. The parameter estimation with *Si* is shown by $\circ$ marker and parameter estimation with *Mu* is shown by * marker. **a**–**c** Plot between nominal values of $T_1$ in x-axis and estimated mean $T_1$ value and standard deviation with error bars **a** from a slice near bottom **b** near center and **c** upper part of the phantom. **d**–**f** shows similar plots for $T_2$. **g** shows the mean and standard deviation with error bar of estimated $T_1$ values across one of the tubes of the phantom. **h** shows the same for $T_2$

## Results

### Phantom experiment I

Figure 2a–c shows the mean $T_1$ as function of nominal $T_1$ for three different slices at different locations along the volume: slices 15 (bottom), 22 (central), and 27 (top). In the central slice (Fig. 2b), the mean $T_1$ values are clustered together for each tube and are at a similar distance from the nominal values represented by the line. In the bottom and top slices (Fig. 2a and c), the *sRF-SENSE* results (red markers) are further away from the nominal values, demonstrating slice dependency due to the slab-selective RF pulses. To further illustrate this, Fig. 2g plots the mean and standard deviation against the slice numbers of one tube in the phantom with a nominal value of 1068 ms. Here, we can see that the red markers representing *sRF-SENSE* show slice dependency. The values in the central part of the tube are closest to the nominal values, while the values diverge from the nominal values at either end of the tube. This is the case both for the *Si* as well as the *Mu* dictionary, though, in the central region, the slice dependence of the *Mu* dictionary that takes the slice dependent effects of the RF pulses into account is substantially lower than the *Si* dictionary. For *nsRF-SENSE* approaches (blue markers), the slice dependency is substantially reduced. Between *Si* (o marker) and *Mu* (* marker), the *Mu* *markers are are closer to the nominal values.

Figure 2d–f shows the results for $T_2$ mapping. The $T_2$ results for the central slice (e) are similar in all four cases. However, the *sRF-SENSE* (red) results are different in top and bottom slices (Fig. 2d and f) showing slice dependency. This has also been highlighted in Fig. 2h, where mean and standard deviation across one tube in the phantom with a nominal value of 212 ms have been shown. Unlike the $T_1$ results, we only observe slice dependency in the case of *sRF-SENSE-Si*. In *sRF-SENSE-Mu*, the slice dependency has been compensated effectively, reducing the slice dependency to a similar level of that of the *nsRF-SENSE* approaches.

**Fig. 3:**
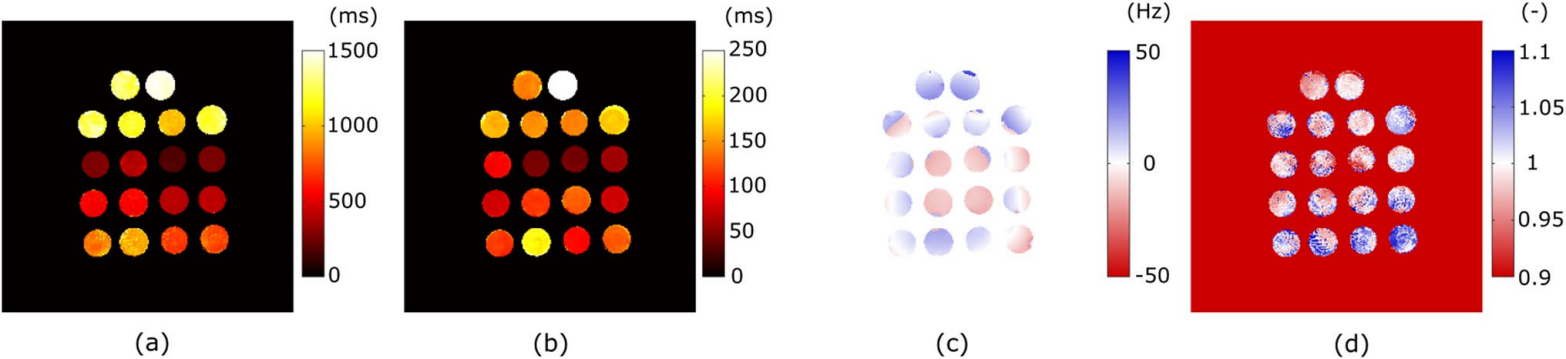
Results from phantom experiment I: QMRI maps generated using *nsRF-SENSE-Mu* **.** a $T_1$ map in milliseconds, **b** $T_2$ map in milliseconds, **c** $B_0$ map in Hertz, and **d** $B_1$ map on a scale where 1 represents $180^\circ$

Figure 3 shows the results of *nsRF-SENSE-Mu* inside a mask that selects the tubes. The parameters are set to zero outside this mask. Visual inspection shows no undersampling or artefacts in $T_1$ and $T_2$. The $B_0$ map in (c) shows some wrap around artefacts within the tubes. The $B_1$ map (d) is not homogeneous in the tubes.

### Phantom experiment II

**Fig. 4:**
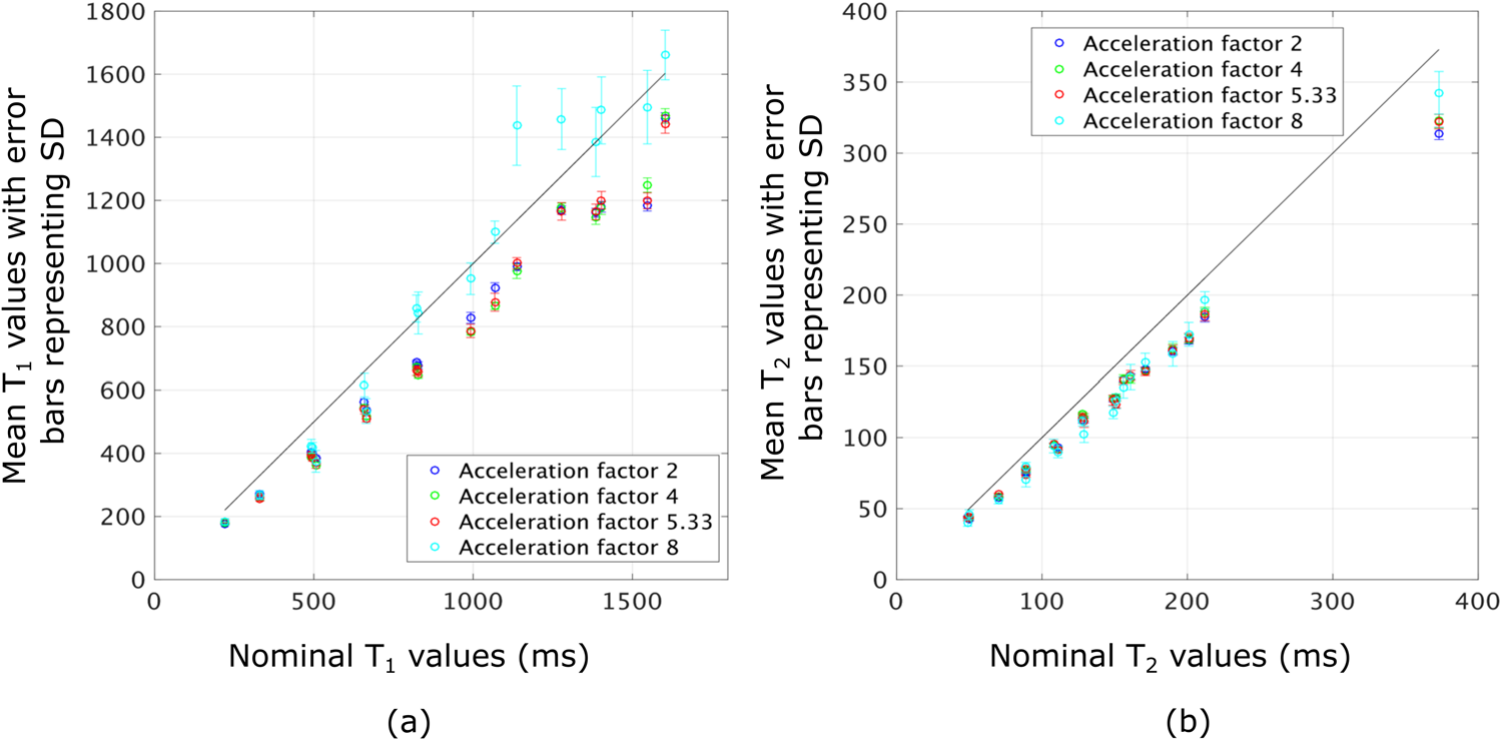
Results from phantom experiment II. **a** Plot between nominal values of $T_1$ in *x*-axis and estimated mean $T_1$ value and error bars with the standard deviation for scans. Similarly, **b** shows the same results for $T_2$

Figure 4a and b shows the ROI statistics of $T_1$ and $T_2$ in the central slice for various acceleration factors obtained with *nsRF-SCR-Mu*. Note that, across all acceleration factors, there is an underestimation for both $T_1$ and $T_2$ in all ROIs compared to the nominal values. The results for acceleration factors up to 5.33 are similar to acceleration factor 2.

### Phantom experiment III

**Fig. 5:**
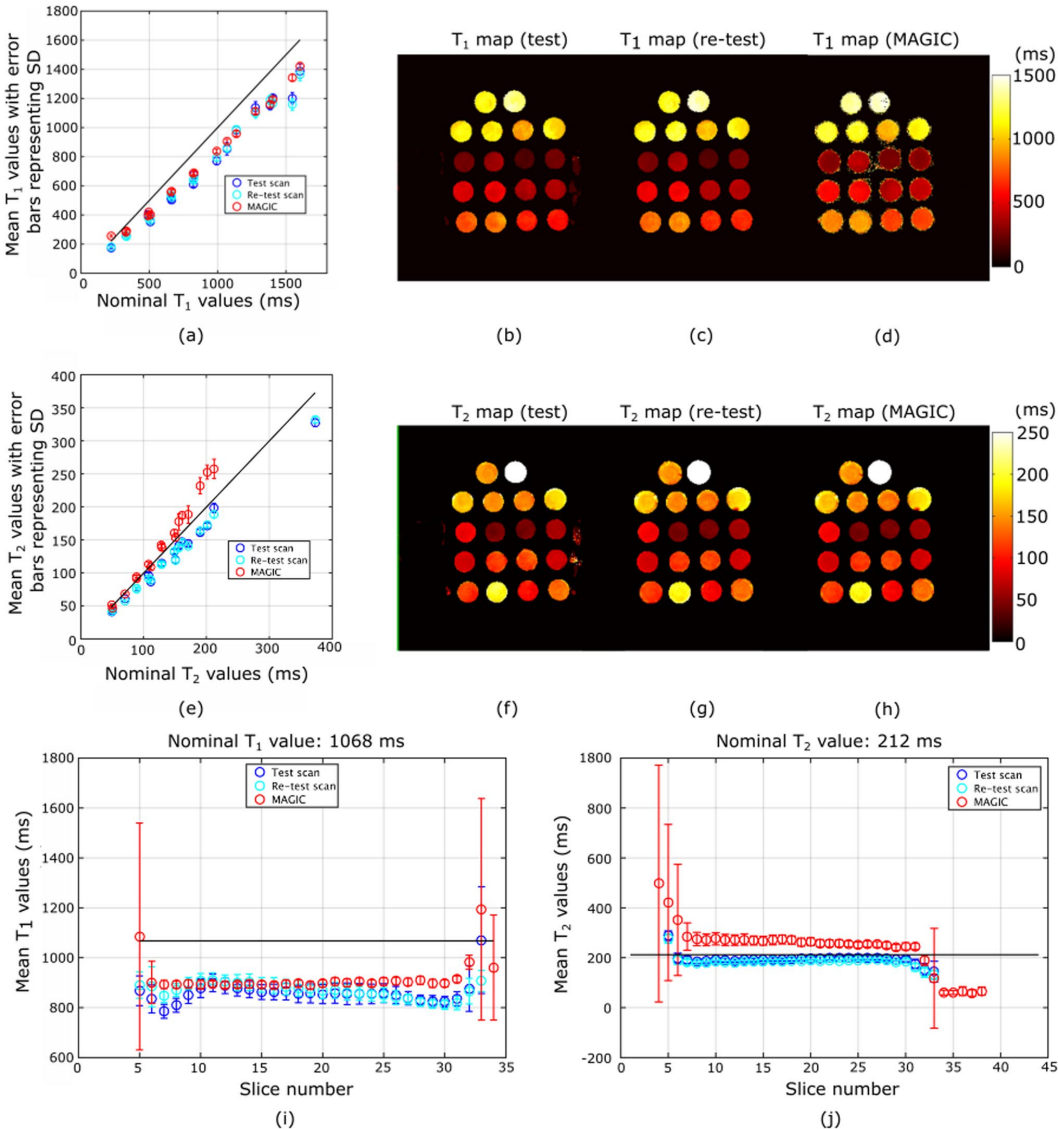
Results from phantom experiment III. **a**, **e** The mean and standard deviation of **a** $T_1$ and **e** $T_2$ inside the ROIs of the central slice (slice 22) in *y*-axis and the corresponding nominal values in *x*-axis. **b**–**d** The axial view of the $T_1$ map from **b** test scan, **c** retest scan, and **d** MAGIC scan. **f**–**h** The axial view of $T_2$ maps from **f** test scan, **g** retest scan, and **h** MAGIC scan. **i** The mean and standard deviation of estimated $T_1$ values across one of the tubes of the phantom, showing the change if any across the slice encoding direction, **j** the same for $T_2$

Figure 5 shows the $T_1$ and $T_2$ values for all tubes obtained from 3D MP-b-nSSFP based on nsRF-SCR-Mu (acceleration factor 5.33) and MAGIC scans. On visual inspection, the maps obtained in the test–retest scan from 3D MP-b-nSSFP look similar to those obtained from MAGIC. Both the mean $T_1$ as well as the mean $T_2$ values obtained from 3D MP-b-nSSFP and MAGIC across all the ROIs were at a similar distance from the nominal value. The difference was less than 30 ms for ROIs within the $50 \le T_2 \le 200$ ms range. Figure 5i, j shows the $T_1$ and $T_2$ values across the slices of a tube. The 3D MP-b-nSSFP shows some slice dependency for $T_1$, while MAGIC does not show such dependency. For $T_2$, the values estimated from 3D MP-b-nSSFP are less slice dependent and are closer to the reference value than those estimated from MAGIC for slice 7–30.

**Fig. 6:**
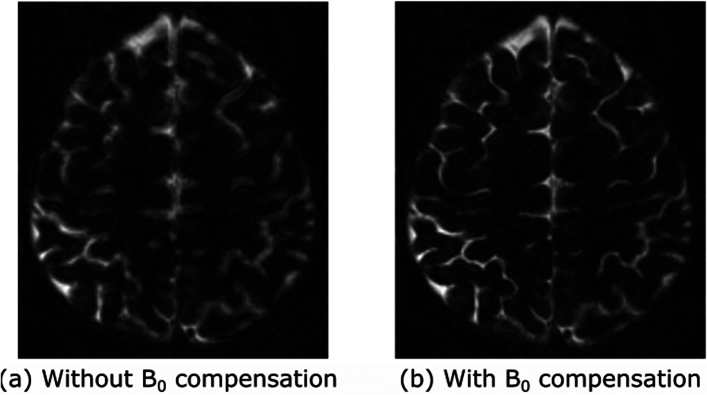
Results from volunteer experiment. Image $q=20$ of nsRF-SCR-Mu, reconstructed without and with $B_0$ compensation

**Fig. 7:**
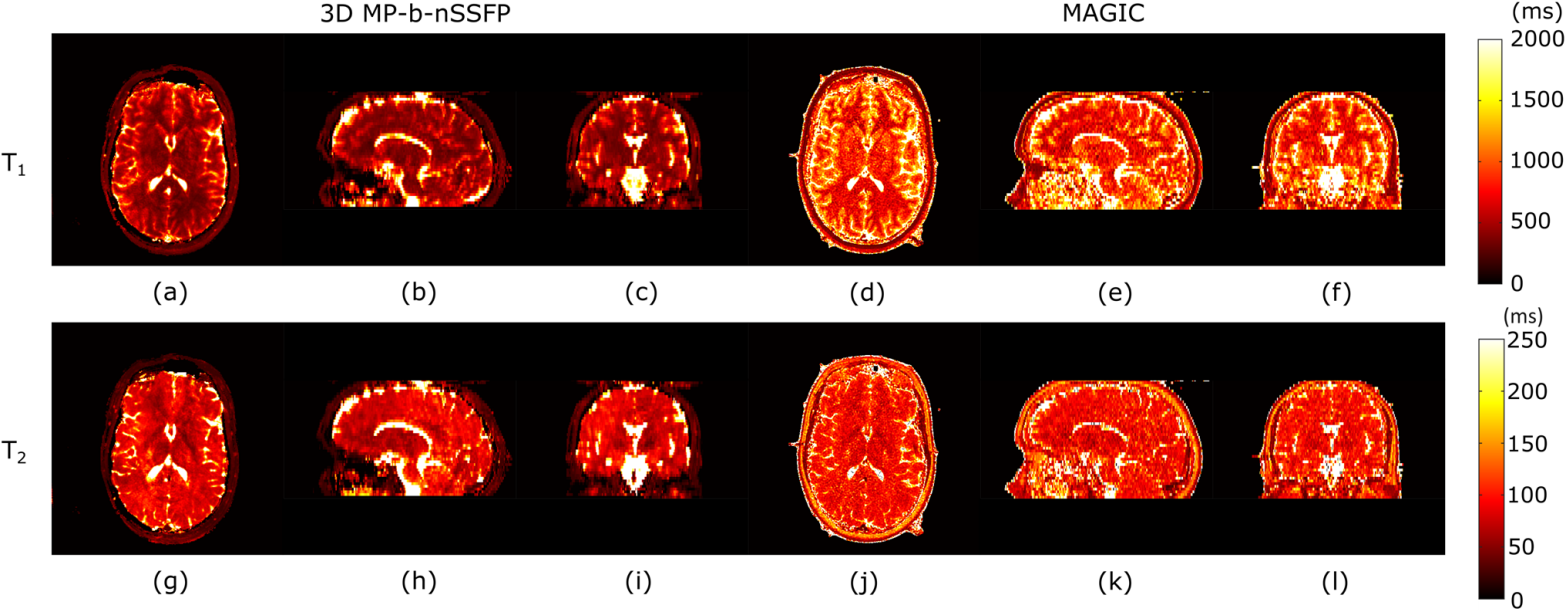
Results from volunteer 1 scan. Axial (**a**, **d**, **g**, **j**), sagittal (**b**, **e**, **h**, **k**), and coronal (**c**, **f**, **i**, **l**) views of the $T_1$ map (**a**–**f**) and $T_2$ map (**g**–**l**) maps of the in vivo 3D MP-b-nSSFP scan nsRF-SCR-Mu with acceleration factor 5.33 (**a**–**c**, **g**, **h**) and the reference MAGIC scan (**d**–**f**, **j**–**l**)

**Fig. 8:**
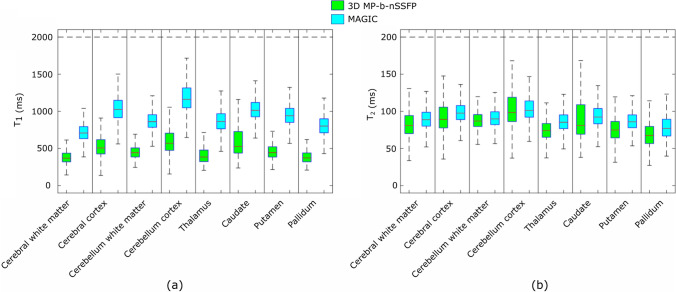
Results from volunteer experiment. The box plot showing the values of **a** $T_1$ and **b** $T_2$ from 3D MP-b-nSSFP based on *nsRF-SCR-Mu*, and MAGIC scans

### Volunteer scans

#### Volunteer scan experiment I

Figure 6 shows one of the reconstructed contrast images, where there are clear blurring artefacts present in reconstruction without $B_0$ compensation step, which are not present in the reconstruction that includes the $B_0$ compensation. Figure 7a–f shows the $T_1$ maps obtained from volunteer 1 scan acquired with 3D MP-b-nSSFP based on *nsRF- SCR-Mu* with acceleration factor of 5.33 and MAGIC. Note that the anatomical structure is clearly depicted without significant blurring artifacts, though the noise appears to have stronger low spatial frequency components. For $T_1$, there is clear distinction between grey matter, white matter, and *CSF* and quite homogeneous values inside the tissues. Similarly, Fig. 7g–l shows the $T_2$ map for the same volunteer. Upon visual inspection, the $T_2$ values of 3D MP-b-nSSFP are in similar range as MAGIC. Figure 8 shows box plot of the $T_1$ and $T_2$ values obtained with 3D MP-b-nSSFP and MAGIC. However, note that, in most regions, the spread of values in the ROI (height of the boxes) is lower for 3D MP-b-nSSFP at this acceleration factor than MAGIC, indicating a lower noise level in the maps. The plot includes eight regions of interest based on the automatic segmentation. The $T_2$ values appear slightly below those of MAGIC, with larger spread in some ROIs. The large error bars for $T_2$ seen in Fig. 8 in the cerebral cortex, cerebellum, and caudate region could be due to misalignment during segmentation. Visual inspection of these ROIs identified several voxels that based on $T_2$ value appear to already be in the bordering CSF but which are included in these brain regions. Still for the majority of ROIs, the boxes overlap. Similar maps and boxplots from two other volunteers can be found in Appendix 4.

#### Volunteer scan experiment II

**Fig. 9:**
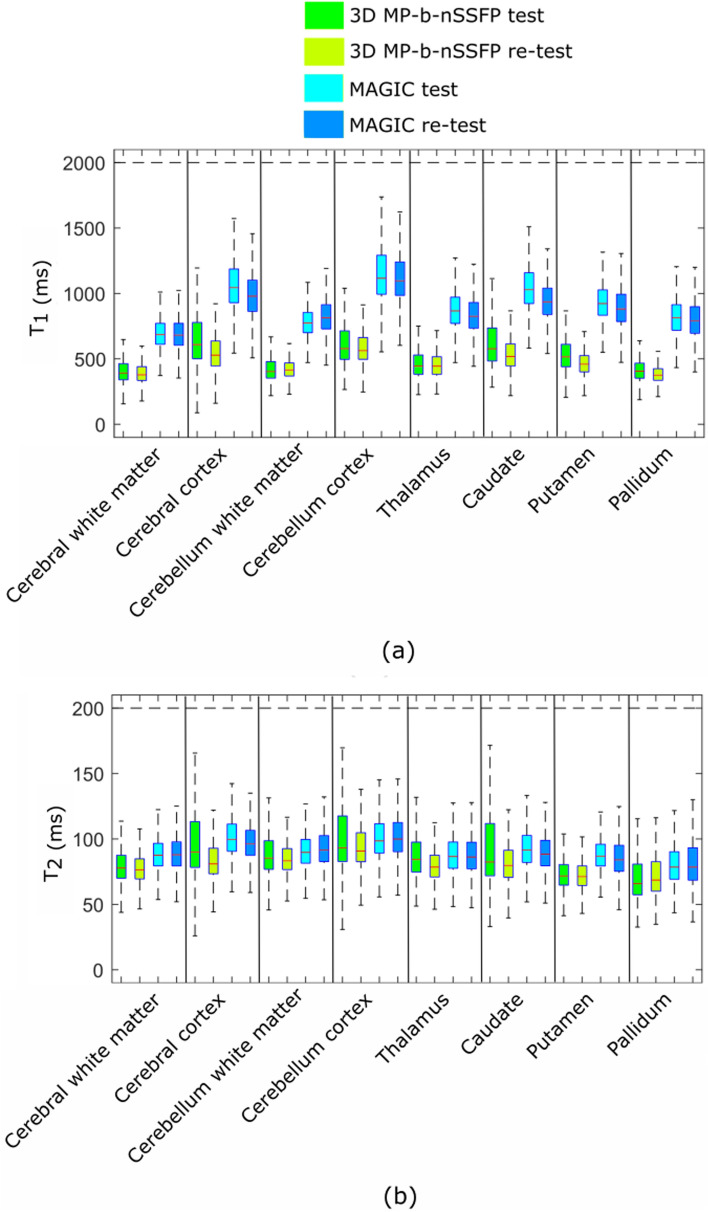
Results from volunteer experiment II. The box plots of values of **a** $T_1$ and **b** $T_2$ from different ROIs obtained from test and retest scan of 3D MP-b-nSSFP and MAGIC

Figure 9 shows the box plot of $T_1$ and $T_2$ maps obtained from test and retest scan using 3D MP-b-nSSFP and MAGIC. Note that the boxes for test and retest overlap in all the ROIs for 3D MP-b-nSSFP and MAGIC.

## Discussion

We have implemented an optimized 3D MP-b-nSSFP for multi-parametric mapping. We compared the resulting 3D scan with phantom and in vivo experiments.

In volunteer scans, we observed similar $T_2$ values for 3D MP b-nSSFP and the reference MAGIC scan in the quantitative analysis. Visually the noise in the $T_2$ maps of 3D MP b-nSSFP had more low spatial frequency components than the reference, possibly due to different motion sensitivity.

Our results show that for this adaptation, using a non-selective RF pulse is better than a slab-selective inversion pulse. Even after modeling, the slice dependency effect in the post-processing pipeline, using a slab-selective inversion pulse, resulted in slice dependent parameter values. We incorporated multi-spin per voxel in the dictionary (*Mu*), which showed $T_1$ maps closer to the nominal values of the phantom in comparison to *Si* which shows that the inclusion of multi-spin per voxel assumption is necessary for accurate $T_1$ mapping with 3D MP-b-nSSFP.

The visual inspection of the $B_1$ map obtained from the phantom scan appeared unrealistic, as $B_1$ maps are generally smooth and lack structural information; this was not the case in the 2D MP-b-nSSFP. The changes in the acquisition introduced for making the sequence 3D made the $B_1$ challenging to estimate from the resulting signal, which needs further investigation.

Although the multiple-spin dictionary approach significantly improves the accuracy of $T_1$ estimates compared to single-spin modeling, particularly around the central slices (as demonstrated in Fig. 2h), notable biases remain in the presence of slab-selective inversion pulses. Interestingly, $T_2$ values obtained with the slab-selective sRF-SENSE-Mu approach closely match those from non-selective (nsRF) inversion, suggesting that the slab-profile effect predominantly affects $T_1$ estimation. The exact cause of the persistent bias in $T_1$ is unclear and was unexpected. One potential explanation is residual mismatches between simulated and experimentally realized RF and gradient pulse shapes, despite careful modeling efforts. Such subtle discrepancies may lead to inaccuracies in capturing slab-profile effects within the dictionary-based matching framework. Future work should carefully investigate this aspect, possibly incorporating more sophisticated pulse calibration or adaptive dictionary refinements to address these residual errors.

We studied the repeatability of 3D MP-b-nSSFP with extended readout and acceleration of 5.33 with test–retest scans and compared it with reference MAGIC scans. In the in vivo repeat scan, MAGIC was considered the reference as previously the in vivo $T_1$ and $T_2$ values were found to be within $6\%$ of gold standard techniques, except for CSF [55]. Both $T_1$ and $T_2$ showed good repeatability with mean $T_1$ and $T_2$ of test and retest being within the standard deviation for each other in majority of the cases. In the phantom, 3D MP-b-nSSFP underestimated $T_1$ by less than 100 ms compared to MAGIC. The differences with respect to nominal values could be due to other factors such as temperature of the phantom, for which we did not control [58]. In volunteer scans, we observed similar $T_2$ values for 3D MP-b-nSSFP and the reference MAGIC scan. However, we observed an underestimation of $T_1$ values in all ROIs. Similar underestimation of in vivo $T_1$ was observed in the 2D MP-b-nSSFP sequence [3]. While the underestimation of $T_1$ in phantom experiments was noticeable, it was less strong than in the in vivo experiments. A possible explanation for this discrepancy between phantom and in vivo results is magnetization transfer [59–61]. In bSSFP sequences, it has been previously reported that the signal from tissues with intra-voxel effects, such as magnetization transfer effects, can result in significant signal loss [59–61], biasing $T_1$ estimates while $T_2$ remains unbiased [62]. We have added an simulation experiment in Appendix 2 to show the effect of magnetization transfer on $T_1$ values with Bloch simulation. Future research can investigate if such effects can be included in the simulations for the dictionary. The test–retest scan for a volunteer showed good repeatability even with underestimation of $T_1$.

In Fig. 3, we can see that noise and artifacts present in the estimated $B_1$ map correlate with the noise pattern in the $T_1$ map, indicating that the $B_1$ is not sufficiently independent from the estimation of other parameters, especially $T_1$. As $B_1$ is spatially varying, using a fixed value introduces a slight, spatially varying bias in the $T_1$ estimates, while estimating $B_1$ on a voxel-wise basis increases the noise level and potentially enhances sensitivity to artifacts.

To reduce the scan time required for 3D scans, we investigated the use of undersampling and extended readouts. We employed subspace-constrained reconstruction to leverage redundancy across signal evolution dimensions and generate parameter maps from undersampled scans. The 12 subspace components used during the reconstruction could describe $99\%$ of the variation in the dictionary, highlighting the compressibility. In the phantom experiment, we found that up to an acceleration factor of 5.33, the accuracy of $T_1$ and $T_2$ is comparable to that of scans reconstructed with acceleration factor 2. We also showed that introducing a $B_0$ compensation step in the reconstruction process reduces blurring and hence enables the extended readout.

The resolution of the scans could be improved by acquiring a large part of k-space in-plane or through-plane. A major obstacle is the increased scan time required for additional measurements. To further reduce scan time, it is worth exploring undersampling the slice encoding direction, which remained fully sampled in our current work. When using a large slab size and suitable multi-channels coils, undersampling the slice encoding direction could achieve further acceleration. With the acceleration factor of 5.33, we have three spirals for every echo. To go to a lower number of spirals, we could extend the length of the spiral readouts even further, which was limited due to the hardware limits. In this work, we focused on exploiting available redundancy in the sequence and chose not to include other prior information in the reconstruction as our hypothesis was that the redundancy across the contrasts would be sufficient. When increasing the resolution, the decreasing voxel volume reduces the intrinsic SNR, which likely limits the achievable acceleration factor. Adding spatial prior information such as provided by compressed sensing techniques (total variation) or data based techniques [63] could help to improve reconstruction quality.

Different from MR fingerprinting, our approach started from analytical definition of the transient response. Even though the method now uses dictionary-based pattern matching similar to MR fingerprinting, we could use analytical fitting like other conventional MR parameter mapping techniques. We opted for a dictionary-based approach primarily because it more flexibly allowed inclusion of the slab profile and was computationally faster in the current implementation.

The original 2D sequence was selected based through Cramér-Rao Lower Bound (CRLB) analysis. While the current study employs a multi-spin simulation that differs from the original signal model, the primary distinction lies in variations in the effective flip angle ($B_1$) across multiple spins. Because the original CRLB analysis accounted for these $B_1$ variations when choosing the sequence blocks, we expect that those blocks will remain appropriate for use in the present study as well, though some optimization might be possible. Similarly to the original work, the 3D scan sequence includes significant dead time, impacting overall scan efficiency. The current 3D sequence involves 12 repetitions of 4-pulse blocks, taking 1440 ms, followed by a 3000 ms delay, leading to approximately $67\%$ recovery time that is not used for imaging. While the delay ensures sufficient magnetization recovery, it limits scan efficiency. We recognize the need to optimize this process to reduce time in which no data is collected.

These delays are essential for complete spin relaxation and signal integrity but highlight a limitation in scan efficiency. We recognize the need to optimize this process to reduce dead time. Future improvements could involve exploring alternative pulse sequences or refining timing parameters to enhance scan efficiency without compromising signal quality.

The $B_0$ compensation step used in this work is similar to that proposed by Dong et al. [64], where SCR is combined with the $B_0$ compensation step. However, since we do not use a calibration region in our acquisition required for their approach, we proposed to obtain the $B_0$ map in a first step of the reconstruction.

## Conclusion

We presented accelerated 3D MP-b-nSSFP that can perform multi-parameter mapping in clinically acceptable scan time. We found that using a non-selective inversion pulse in the slice encoding dimension had a lower bias in $T_1$ and $T_2$ than using a slice-sensitive inversion pulse and modeling its slice position dependent behavior. The mean $T_1$ values obtained from 3D MP-b-nSSFP phantom scans were within 100 ms from those obtained from MAGIC. However, we found an underestimation of $T_1$ in the volunteer scans, which needs further investigation. The accuracy of $T_2$ from 3D MP-b-nSSFP was as good as the reference technique in both phantom and volunteer scans. 

## Figures and Tables

**Fig. 10:**
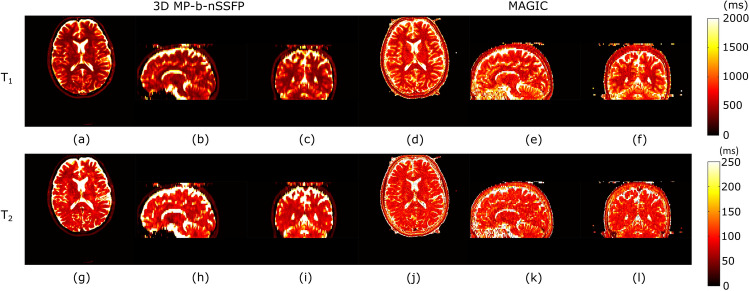
Results from volunteer 2 scan. Axial (**a**, **d**, **g**, **j**), sagittal (**b**, **e**, **h**, **k**), and coronal (**c**, **f**, **i**, **l**) views of the $T_1$ map (**a**–**f**) and $T_2$ map (**g**–**l**) maps of the in vivo 3D MP-b-nSSFP scan nsRF-SCR-Mu with acceleration factor 5.33 (**a**–**c**, **g**, **h**) and the reference MAGIC scan (**d**–**f**, **j**–**l**)

**Fig. 11:**
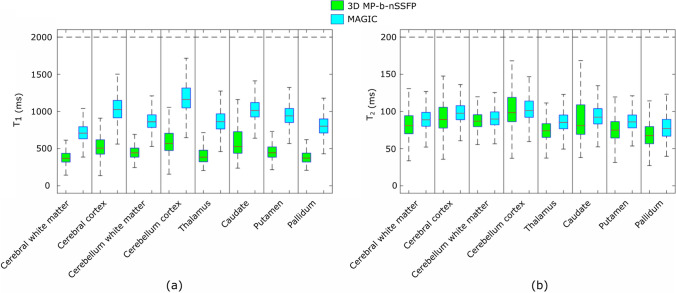
Results from volunteer experiment. **a**, **b** The box plot showing the values of **a** $T_1$ and **b** $T_2$ from 3D MP-b-nSSFP and MAGIC scans

**Fig. 12:**
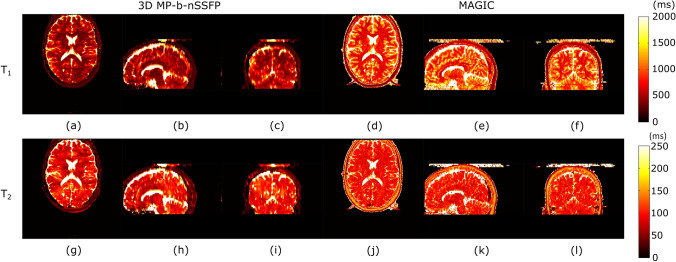
Results from volunteer 3 scan. **a** Axial, **b** sagittal, and **c** coronal view of $T_2$ map obtained using 3D MP-b-nSSFP scan with acceleration of 5.33. **d** Axial, **e** sagittal, and **f** coronal view of $T_2$ map (ms) obtained using MAGIC scan. **g** Axial, **h** sagittal and **i** coronal view of $T_2$ map obtained using 3D MP-b-nSSFP scan with acceleration of 5.33. **j** Axial, **k** sagittal and **l** Coronal view of $T_2$ map (ms) obtained using MAGIC scan

**Fig. 13:**
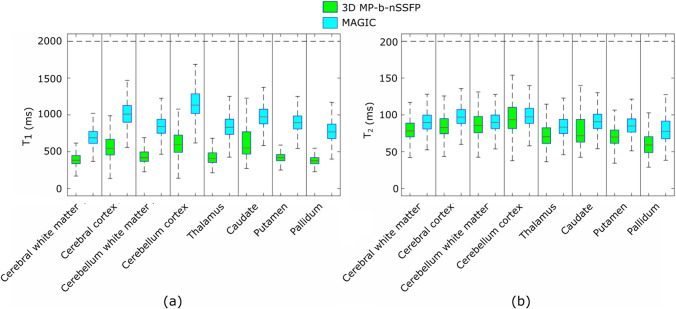
Results from volunteer experiment. **a**, **b** The box plot showing the values of **a** $T_1$ and **b** $T_2$ from 3D MP-b-nSSFP and MAGIC scans

**Fig. 14:**
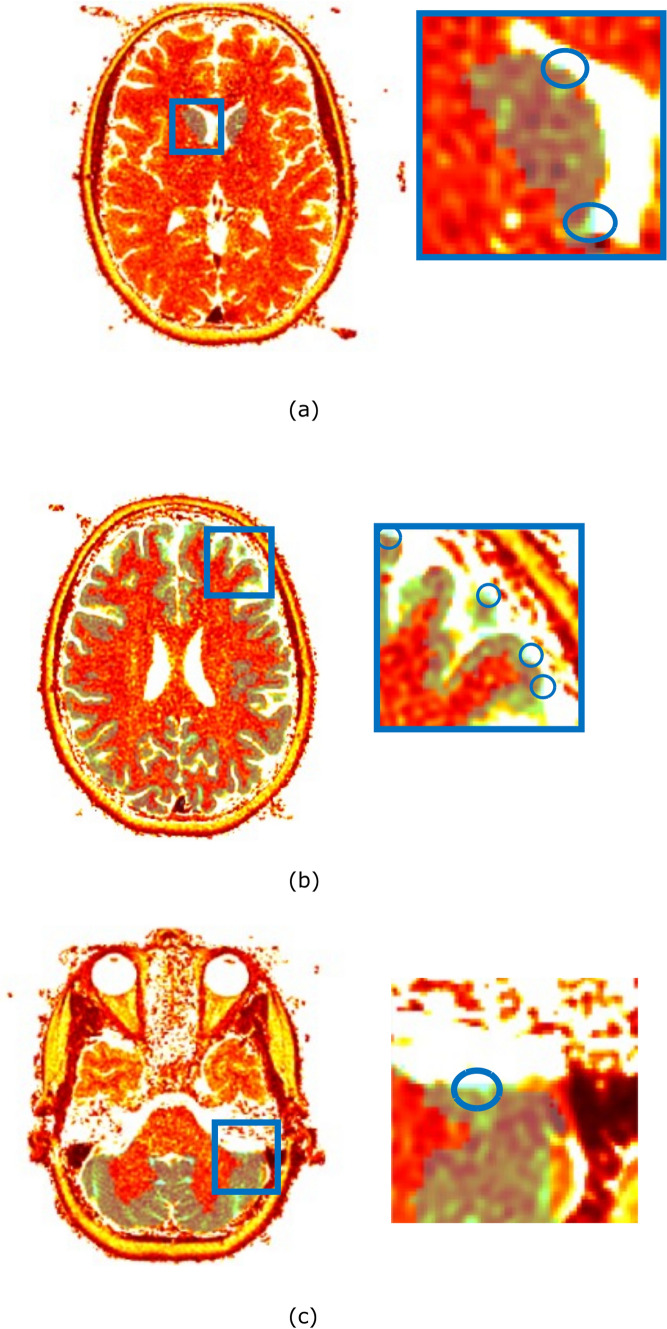
$T_2$ maps of the cerebral cortex (**a**), cerebellum cortex (**b**), and caudate (**b**) regions. The masks for the ROI analysis are shown as cyan/green-colored overlay. Clearly illustrating alignment and coverage on these brain structures. Zoomed-in views (right panels) are provided to detail mask accuracy and anatomical specificity within each ROI

## Data Availability

The data that support the findings of this study are not openly available due to reasons of sensitivity and are available from the corresponding author upon reasonable request. Data are located in controlled access data storage at Erasmus MC.

## Appendix 1: $B_{0}$ map unwrapping

We used the following ‘ad hoc’ 3D unwrapping procedure which worked for the evaluated acquisitions, but more optimal solutions likely are possible. Specifically, we used an iteratively re-weighted third-order polynomial fit to the $B_0$ map to unwrap it. Let $\varvec{R}$ represent the general third degree polynomial expression and $\varvec{\Delta }_{in} = \tfrac{2 \pi \varvec{B_0}}{\text {TR}}$ represents the $B_0$ map with phase wraps represented in phase cycles per TR. We use the following algorithm.

Let $\otimes$ represent elementwise multiplication (Hadamard product), $\times$ represent matrix multiplication, $\lfloor \rceil$ represent rounding operation and regression basis be represented by $$\varvec{R} = \begin{bmatrix} X^{3},&3YX^{2},&3ZX^{2},&3XY^{2},&6XYZ,&Y^{3},&3ZY^{2},&3YZ^{2},&Z^{3} \end{bmatrix}$$

Initialization:

   $$w \leftarrow |\varvec{I_0}|$$

   $$\varvec{\beta } \leftarrow \varvec{\Delta }_{in}$$

Loop over *t* iterations:

(i) $$\varvec{\beta }^{fit} \leftarrow \frac{\varvec{R} \times {\left( w \otimes \varvec{\beta }\right) }}{w \otimes \varvec{R}}$$
(ii) $$\varvec{\beta } \leftarrow \varvec{\beta } - \lfloor \left( \varvec{\beta } - \varvec{\beta }^{fit}\right) \rceil$$
(iii) $$w \leftarrow |\varvec{I_0}|e^{\left( \varvec{\beta }^{fit} - \varvec{\beta }\right) ^{2k}}$$

Apply Gaussian filter with sigma of 1 in all three dimensions to get the $B_0$ map which is converted to Hz:

$$\varvec{\Delta }_{est} \leftarrow G(\varvec{\beta }) \frac{TR}{2\pi }$$

where function *G* applies Gaussian filter.

## Appendix 2: Sensitivity to magnetization transfer (MT) effects

This simulation experiment aims to check the sensitivity of 3D MP-b-nSSFP, and especially the $T_1$ estimated from it, to MT effects. We performed a two pool Bloch simulation according to a custom implementation done by Cabana et al. [65], using the exact RF waveforms and crusher gradients as present in the pulse sequence. We simulate the signal of four voxels using a two pool Bloch simulation of the 3D MP-b-nSSFP sequence. The voxels differed only in the restricted pool size (MT fractions) as shown in Table 2. The qMRI values were then estimated using dictionary matching technique with dictionary *Mu* described in Sect. 3.

Table 2 shows the ground truth and estimated parameter values. The estimated $T_1$ decreases with increasing MTF, while the other parameters show little deviation from the ground truth, except for a constant scaling of the PD value due to different signal scaling in the two pool simulation with respect to the multi-spin simulation. This shows that in 3D MP-b-nSSFP, MT can induce a substantial bias on, mainly, the $T_1$ estimate.

**Table 2:** Table shows results from qMRI parameters estimated from the simulation experiment with four MT fractions

Parameters	Ground truth	Estimated values for MTF			
		0.001	0.05	0.075	0.12
$T_1$ (ms)	1000	971	736	349	244
$T_2$ (ms)	100	100	99	99	100
$B_0$ (Hz)	$-$ 1.6	$-$ 1.62	$-$ 1.58	$-$ 1.56	$-$ 1.51
PD	1	2.43	2.44	2.45	2.45

## Appendix 3: Remaining volunteer scans

Figure 10 shows $T_1$ and $T_2$ map from volunteer 2 and Fig. 11 shows the box plots that compares $T_1$ and $T_2$ map to those from MAGIC in respective ROIs. Similarly, Figs. 12 and 13 show those from volunteer 3.

## Appendix 4: Misalignment with segmentation mask

Figure 14 shows misalignment due to segmentation error which causes the error bars to be longer in Fig. 8.

## Appendix 5: Derivation of SCR for $B_0$ inhomogeneity corrected contrast images

In the presence of ${B_0}$ inhomogeneity, let $\varvec{M}^{'}$ be approximated by:

7 $$\begin{aligned} {M}^{'}_{q,v,p} = {M}_{q,v} e^{-i{\Delta }_{v}\tau _{p}}. \end{aligned}$$

where $\tau _{p}$ is the time offset with respect to magnetization state ${M}^{'}_{q,v,p}$. Substituting ${\sigma }_{ m, v} = {{S}_{m,m}} {{V^{H}_{v,m}}}$ in Eq. 2 gives:

8 $$\begin{aligned} \varvec{M} \approx \varvec{U} \varvec{S} \varvec{V^{H}} = \left[ \sum _{m} {{U}_{q, m}} {\sigma }_{ m, v} \right] _{q, v}. \end{aligned}$$

Subspace-constrained reconstruction to estimate $\varvec{{M}^{'}}$ can be defined as:

9 $$\begin{aligned} \hat{\varvec{{M}}}^{'} = \arg \min _{\varvec{{M}^{'}}} \sum _{q,c,p} \Vert {{Y}_{q,c,p} - \sum _{v} \mathcal {F}_{q,p,v} {C_{c,v}} {M}^{'}_{q,v,p} } \Vert ^2 . \quad \end{aligned}$$

Substituting the value of ${M}^{'}_{q,v,p}$ from Eq. 7 in Eq. 9 gives:

10 $$\begin{aligned} \hat{\varvec{M}} = \arg \min _{\varvec{M}}\sum _{q,c,p} \Vert {{Y}_{q,c,p} - \sum _{v} \mathcal {F}_{q,p,v} {C_{c,v}} {M}_{q,v} e^{-i{\Delta }_{v}\tau _{p}}} \Vert ^2 . \quad \end{aligned}$$

Substituting the values of $\varvec{M}$ and $\left[ e^{-i\varvec{\Delta }_{v}\tau _{p}} \right] _{p, v}$ from Eqs. 8 and 4, respectively, in Eq. 10, we get:

11 $$\begin{aligned} \hat{\varvec{\sigma }} = \arg \min _{\varvec{\sigma }} \sum _{ q, c, p} \Vert {Y}_{ q, c, p} - \sum _{ m, w, v} {\tilde{U}}_{ p, w} {\tilde{S}}_{ w, w} U_{ q, m} \mathcal {F}_{q, p,v} \varvec{C}_{c, v} \tilde{V}^{H}_{ v, w} {\sigma }_{ m, v} \Vert ^2 . \quad \end{aligned}$$
